# Esketamine pretreatment during cesarean section reduced the incidence of postpartum depression: a randomized controlled trail

**DOI:** 10.1186/s12871-023-02398-1

**Published:** 2024-01-10

**Authors:** Shixia Xu, Jiaojiao Yang, Jing Li, Min Zhang, Jie Sun, Qingren Liu, Jianjun Yang

**Affiliations:** 1 Department of Anesthesiology, Nanjing Medical University, Nanjing, 210000 Jiangsu China; 2 Department of Anesthesiology, Zhongda Hospital, School of Medicine, Southeast University, Nanjing, 210000 Jiangsu China; 3 Department of Anesthesiology, Xishan People’s Hospital of Wuxi City, Wuxi, 214105 China; 4 Department of Anesthesiology, Pain and Perioperative Medicine, The First Affiliated Hospital of Zhengzhou University, Zhengzhou, 450000 China

**Keywords:** Cesarean section, Postpartum depression, Esketamine, Parturient

## Abstract

**Background:**

Postpartum depression (PPD) is a common mental disease in postpartum women, which has received more and more attention in society. Ketamine has been confirmed for its rapid antidepressant effect in women with PPD. We speculate that esketamine, an enantiomer of ketamine, pretreatment during cesarean can also reduce the incidence of PPD.

**Methods:**

All the parturients enrolled in the study were randomly assigned to two groups: the esktamine group (0.2 mg/kg esketamine) and the control group (a same volume of saline). All the drugs were pumped for 40 min started from the beginning of the surgery. The Amsterdam Anxiety and Information Scale (APAIS) scores before the surgery, the Edinburgh postnatal depression scale (EPDS) scores at 4 d and 42 d after surgery, the Pain Numerical Rating Scale (NRS) scores at 6 h, 12 h, 24 h and 48 h post-operation were evaluated, as well as the adverse reactions were recorded.

**Results:**

A total of 319 parturients were analyzed in the study. The incidence of PPD (EPDS score > 9) in the esketamine group was lower than the control group at 4 days after surgery (13.8% vs 23.1%, *P* = 0.0430) but not 42 days after surgery (*P* = 0.0987). Esketamine 0.2 mg/kg could reduce the NRS score at 6 h,12 h and 24 h after surgery, as well as the use of vasoactive drugs during surgery (*P* < 0.05). The incidences of maternal dizziness (17.0%), blurred vision (5%), illusion (3.8%) and drowsiness (3.8%) in the esketamine group were higher than those of control group (*P* < 0.05).

**Conclusions:**

Intraoperative injection of esketamine (0.2 mg/kg) prevented the occurrence of depression (EPDS score > 9) at 4 days after delivery but not 42 days. Esketamine reduced the NRS scores at 6 h, 12 h and 24 h after surgery, but the occurrence of maternal side effects such as dizziness, blurred vision, drowsiness and hallucination were increased.

**Trial registration:**

Registered in the Chinese Clinical Trial Registry (ChiCTR2100053422) on 20/11/2021.

## Background

Postpartum depression (PPD) is a common mental disorder in postpartum women, affecting about 10%-15% unipara [1], accounting for about 20% of maternal deaths [2]. It is reported that the incidence of PPD in Chinese is about 22%-28% [3, 4]. PPD affects not only the health of postpartum women, but also their relationships with their children and families, and there is growing evidence show that PPD has harmful effects on the mood and cognition of children in infancy and childhood periods [5]. Mild PPD refers to a mild and tempory illness that occurres 4–6 days after delivery. If the depression state lasts longer than 2 weeks, it is classified as neurotic depression and may require psychotherapy or medication therapy [6]. In most cases, early depression has the risk of transforming into long-term depression [7].

Cesarean section is a common obstetric surgery, accounting for about 25%-45% of all births in China [8, 9]. Cesarean section can lead to a series of physiological changes in the pregnant women, such as infection, postpartum hemorrhage, ureteral and bladder damage, chronic pelvic pain and gastrointestinal dysfunction [10]. These adverse consequences induced by surgical trauma may increase the probability of PPD [11]. Compared with vaginal delivery, women who delivered by cesarean section are more likely to suffer from PPD [12].

Ketamine is a non-selective N-methyl-D-aspartate (NMDA) receptor inhibitor, which has a rapid antidepressant effect and can reduce patients' suicidal intentions in several clinical trials in patients with treatment-resistant depression [13, 14]. Ketamine can also reduce the symptoms of PPD [12]. Esketamine, an enantiomer of ketamine, is twice as potent as ketamine and more than three times as potent as R-ketamine [15, 16]. In February 2019, the US Food and Drug Administration approved esketamine for intranasal treatment of PPD and treatment-resistant Major depressive disorder (MDD) [17]. Basic science research have found that low-dose esketamine can excert an antidepressant effect by enhancing the synaptic plasticity of hippocampus in PPD mice [18]. Clinically, esketamine can improve the functional prognosis of patients with refractory depression [19], and reduce postoperative depression in breast cancer patients without increasing the incidence of adverse events [20].

In summary, this study assumed that 0.2 mg/kg esketamine used during cesarean section might reduce the occurrence of PPD.

## Methods

### Study design

This study was a randomized double-blind single-center trial. It was conducted in Zhongda Hospital Affiliated to Southeast University, approved by the Ethics Committee of Zhongda Hospital Affiliated to Southeast University with the approval number 2020ZDSYLL265-P01 (08/01/2021), and registered on the Chinese clinical trial registration website with the registration number ChiCTR2100053422 (20/11/2021). All patients have signed informed consent.

### Participants

The study was conducted from July 1, 2020 to July 21, 2023, and the last follow-up visit was on September 1, 2023. Inclusion criteria: parturients undergoing a cesarean section with gestational age ≥ 36 weeks, single fetus, abstaining from food and drink, American Society of Anesthesiologists (ASA) grade II, subarachnoid block and epidural catheterization, signed informed consent were included in the study. The exclusion criteria included: (1) mental disease; (2) self-rating Depression Scale (SDS) ≥ 50 points; (3) communication barriers; (4) severe eclampsia; (5) drug or alcohol abuse; (6) esketamine allergy; (7) poor anesthetic effect; (8) change anesthesia method during operation; (9) the fetus has congenital diseases or abnormalities after delivery.

The SDS scale consists of 20 questions with 1–4 points for each question, and the total crude score ranges from 20 to 80 points. Each score value is multiplied by 1.25 to obtain the SDS score. Score below 50 is considered as no depression, 50–59 as mild depression, 60–69 as moderate depression, and a score above 70 as severe depression [21].

### Randomization and blinding

This study was a randomized double-blind trial using a computer randomized number table method conducted by an assistant anesthetic (Jing Li), and the parturients were randomly divided into esketamine group and control group. The group allocation and study drug regimen were hidden in sequentially numbered sealed opaque envelopes. Dispense the medication into a 20 ml syringe and labeled the syringe with the name of the parturient. On the day of cesarean section, the anesthesiologist (Shixia Xu), who was unknown of the study protocol, opened the envelope in the order in which subject was enrolled, and check the name on the syringe one more time. Neither the anesthesiologist nor the follow-up investigator knew the grouping. This information was hidden until the follow-up completed. The follow-up of the study was conducted by Min Zhang. Anesthesiologists couldn't blind of the group allocation because of the possible side effects of esketamine, but the parturients and the follow-up investigator were both blind of the allocation.

### Procedures

The Amsterdam Anxiety and Information Scale (APAIS) score was performed on all parturients before surgery. After entering in the operation room, all parturients were subjected to electrocardiogram, pulse oxygen and non-invasive blood pressure monitoring, and nasal catheter oxygen inhalation was given to 3 L/min. Subarachnoid puncture was performed at L2-3 or L3-4, and 3 ml of 0.5% ropivacaine (2 ml 0.75% ropivacaine + 1 ml 5% glucose) was given in the subarachnoid space. The catheter was placed toward the head of the patient, and the depth of the catheter in the epidural space is 3 cm. After the procedure is completed, the operation bed was tilted 30 degree to the left, the anesthesia level was maintained at T4-T6 level. If a hypotension occured (a systolic blood pressure of below 90 mmhg or lower than 30% of starting systolic blood pressure or MAP less than 70 mmhg), the anesthesiologist would give vasoactive drugs (phenylephrine or ephedrine), to maintain the hemodynamic stable. The pre-configured drugs were given (esketamine 0.2 mg/kg/20 ml or 20 ml saline, 40 min continuous pumping) at the beginning of the operation.

Routine intravenous self-controlled analgesia pump (PCIA) was connected after surgery. Formula: sufentanil citrate injection 2 ug /kg + ondanseetron 16 mg, in a total volume of 100 ml with normal saline. The background rate was 1 ml/h, the bolus dose was 2 ml, with a locked out interval of 15 min, and the extreme limit was 10 ml. All the parturients were taught to use PCIA.

### Outcome measures and data collection

APAIS scores before the surgery, Edinburgh Depression Scale (EPDS) scores at 4 d and 42 d after CS (cesarean section), as well as Pain Numerical Rating Scale (NRS) scores at 6 h, 12 h, 24 h and 48 h postnatal were recorded. APAIS is a self-reported questionnaire used to quickly assessing preoperative anxiety. The scale consists of six items which can be divided into two scales, including an anxiety scale (items 1, 2, 4, and 5) and a need-for-information scale (items 3 and 6) [22]. It is a “gold standard” for preoperative anxiety measurement [23], which could be completed in 2 min, and with a high response rate and good acceptability in Chinese patients as well [24]. EPDS is a widely used self-rating scale consisting of 10 items, based on the severity of symptoms, each content is scored in 4 levels (0, 1, 2, 3 points). The sum of the scores of 10 items is the total score range of 0–30 points, score > 9 is diagnosed as PPD [25, 26]. The NRS score uses the numbers 0–10 instead of words to describe the severe pain in the past 24 h. The numbers 0 is painless, 1–3 is mild pain (does not affect sleep), 4–6 is moderate pain, 7–9 is severe pain (affects sleep), and 10 is severest pain.

Basic information of the parturients was recorded, including height, weight, education, employment, the mode of pregnancy and number of cesarean sections. Basic vital signs, dosage of anesthetic drugs, the use of the vasoactive drugs, anesthesia time, operation time, as well as adverse reactions (including dizziness, headache, nausea, vomiting, blurred vision, hallucination, chest distress, shiver, drowsiness) were recorded.

### Sample size and statistical analysis

The reported rate of PPD in China is 28.2% [3]. We expected using esketamine can reduce the incidence of PPD about 15%, with a statistical power of 80% and α = 0.05 yielded a sample size of 314 (157 in each group). In order to account for a 10% drop-outs, 173 patients were randomly assigned to each group, so we included a total of 346 patients.

Data were analyzed with GraphPad Prism 8 (GraphPad Software Inc., San Diego, CA, USA). Continuous data were expressed using mean with standard deviation, using Student’s t-test or Mann–Whitney U test to compare the differences between 2 groups. Categorical data were expressed using number with percentage and compared between 2 groups using the chi-square test or the Fisher’s exact test. Due to the skewed distribution of depression score, Spearman was used to analyze the correlation between the depression score and other related factors. *P* values less than 0.05 were considered as statistically different.

## Results

**Fig. 1:**
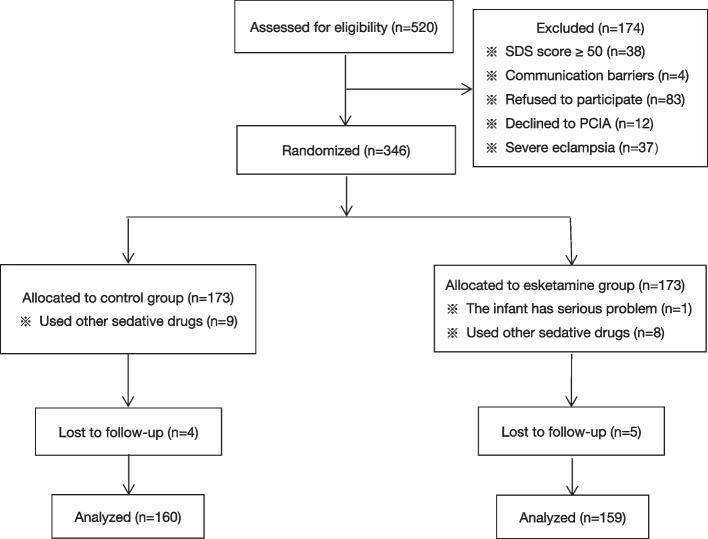
Flowchart of the study. PCIA: patient controlled intravenous analgesia

From July 2020 to September 2023, a total of 520 parturients were included in the study and 174 were excluded, 38 with SDS score ≥ 50 points, 4 had communication barriers, 83 refused to participate in the study, 12 refused to use intravenous analgesia pumps, and 37 had severe eclampsia, 9 parturients in the control group and 8 parturients in the esketamine group used propofol because of a poor anesthesia effect. 1 parturient in the esketamine group delivered a fetus with albinism, 4 parturients in the control group and 5 in the esketamine group lost to 42 d follow-up. Finally, there were 319 parturients included in the analysis, with 160 parturients in the control group and 159 parturients in the esketamine group (Fig. 1).

### Baseline characteristics

There was no significant statistical difference in the basic information of the two groups (Table 1). The average age of the parturients was 30.6 ± 3.8 years old, and the average BMI was 28.4 ± 4 kg/m^2^. Most of the parturients had a bachelor's degree (71.2%), 265 were employed (83.1%), and 225 were uniparas (70.5%). Most were emergency operations (60.5%).

**Table 1:** Baseline characteristics of parturients and surgeries in the two groups

Variables	Control group (*n* = 160)	Esketamine group (*n* = 159)	*P*-value
Age (year)	30.9 ± 3.8	30.3 ± 3.8	0.1398
Height (cm)	161.4 ± 4.4	161.4 ± 4.4	0.4425
Weight (kg)	74.6 ± 11.5	73.6 ± 11.1	0.4252
BMI (kg/m^2^)	28.5 ± 3.9	28.3 ± 4.1	0.6105
APAIS Score			
Total score	12.7 ± 6.1	12.9 ± 5.5	0.7021
Information requirement score	3.9 ± 2.3	4.3 ± 2.5	0.1266
Education level			0.7502
Primary school	1 (0.6%)	0 (0)	
Secondary school	10 (6.3%)	9 (5.6%)	
High school	17 (10.7%)	17 (10.6%)	
Bachelor	110 (69.2%)	118 (73.8%)	
Master	18 (11.3%)	12 (7.5%)	
Doctor	3 (1.9%)	4 (2.5%)	
Employment	131 (81.9%)	134 (84.3%)	0.6546
Prior history of CS	46 (28.8%)	48 (30.2%)	0.8068
Type of operation			0.6477
Emergency	99 (61.9%)	94 (59.1%)	
Selective	61 (38.1%)	65 (40.9%)	
Mode of pregnancy			0.6526
Natural	148 (92.5%)	150 (94.3%)	
Unnatural	12 (7.5%)	9 (5.7%)	
Anticipate			0.8351
Expected	49(30.8%)	42(26.3%)	
Unexpected	31(19.5%)	30(18.7%)	
Indifferent	48(30.2%)	53(3.1%)	
Known	31(19.5%)	35(21.9%)	
Surgery time (minute)	61.9 ± 11.1	59.2 ± 13.8	0.0502
Anesthesia time (minute)	79.8 ± 13.0	77.3 ± 15.6	0.1275

Data are presented as mean ± SD, median (IQR), or number (percentage)

*BMI* body mass index, *APAIS* Amsterdam Anxiety and Information Scale, *CS* cesarean section

### Primary outcome

**Fig. 2:**
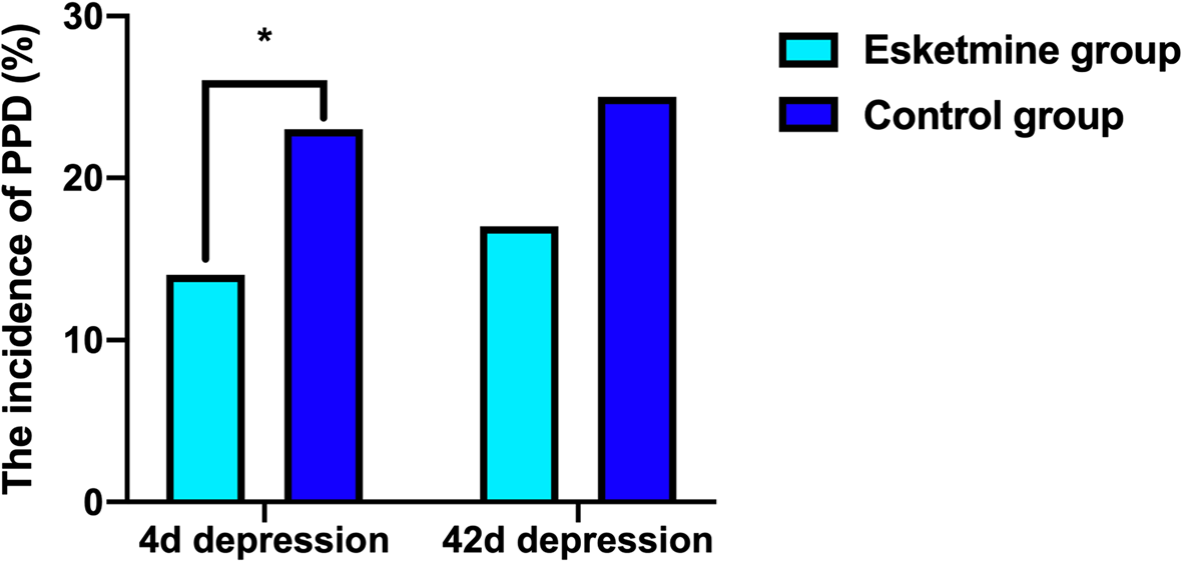
The incidences of postpartum depression (PPD) at 4 d and 42 d in the two groups. *P < 0.05

**Fig. 3:**
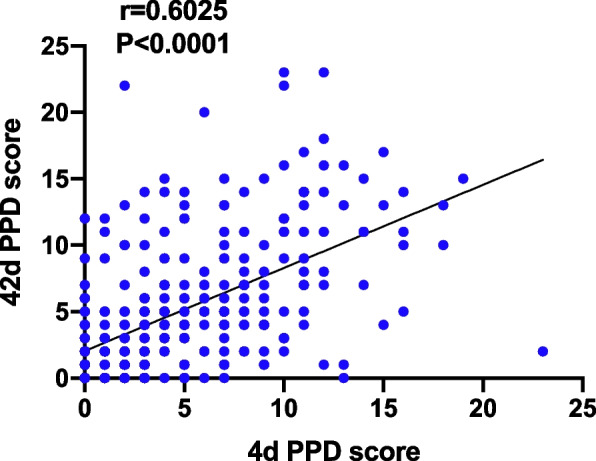
Correlation between postpartum depression (PPD) scores at 4 d and 42 d

**Fig. 4:**
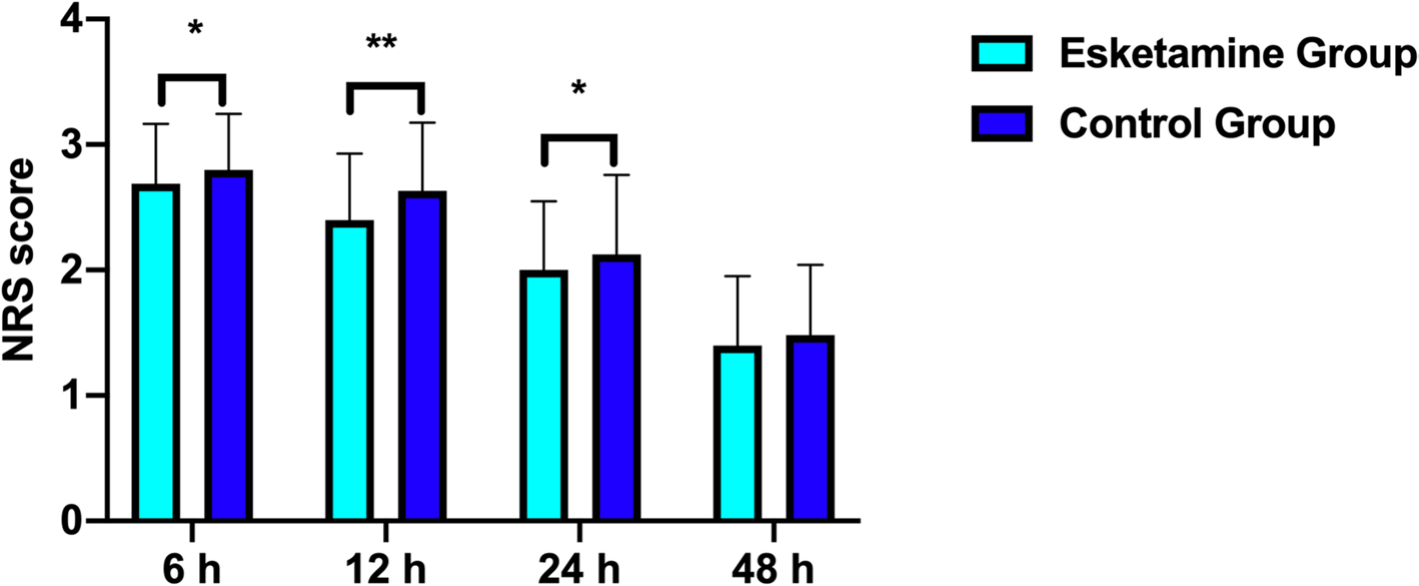
Postoperative numeric rating scales (NRS) scores at 6 h, 12 h, 24 h and 48 h after surgery in the two groups. *P < 0.05; **P < 0.001

**Fig. 5:**
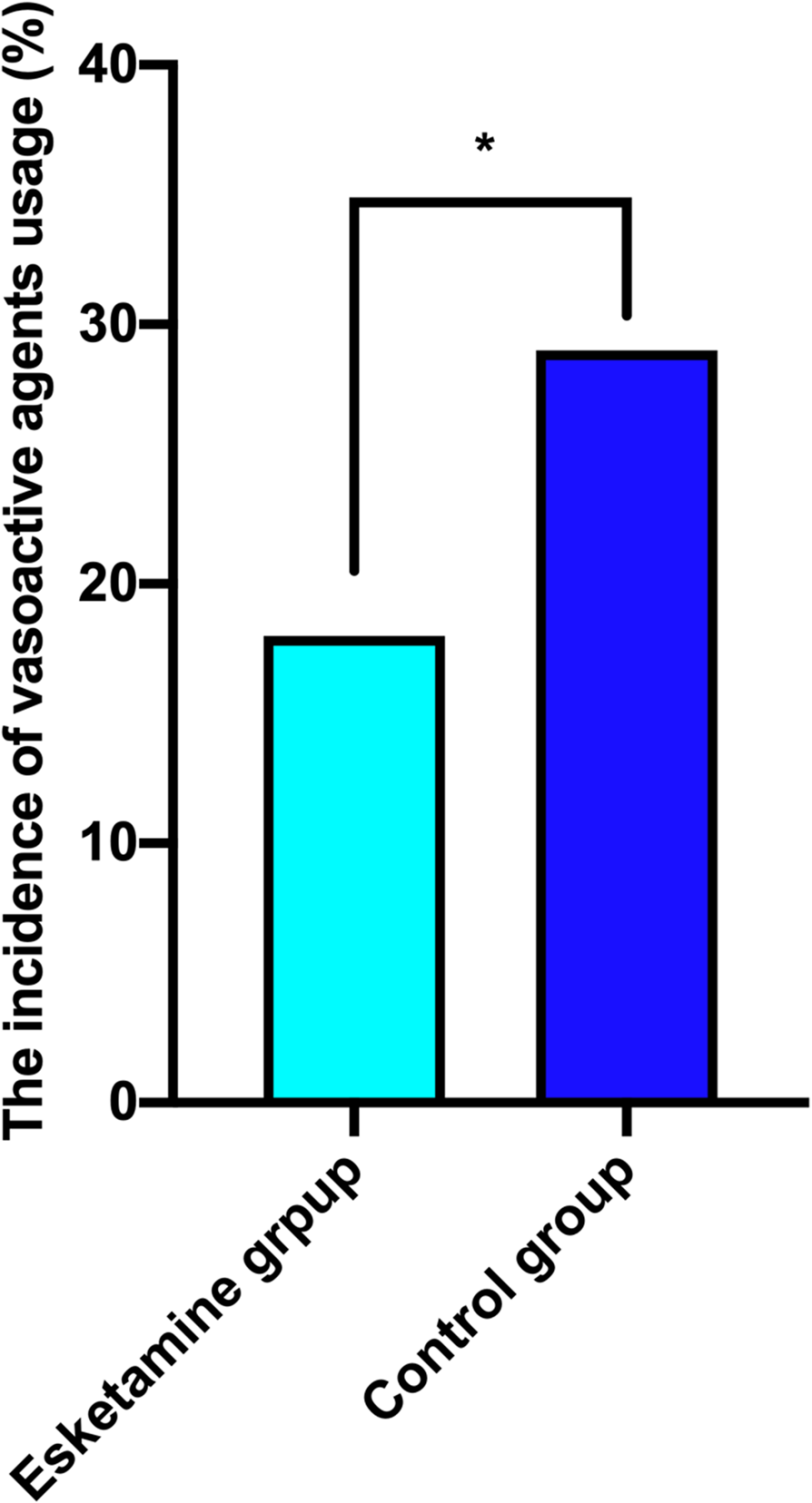
Positive vasoactive agents usage in the two groups. *P < 0.05

**Fig. 6:**
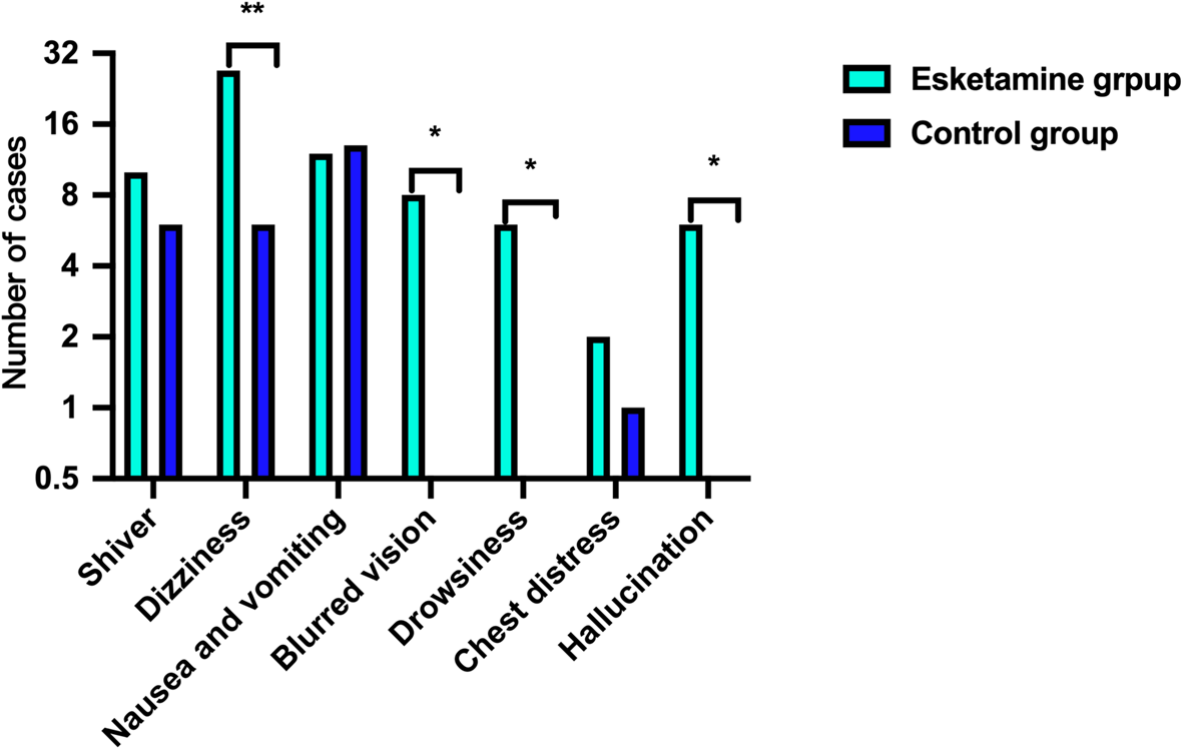
Side effects between the two groups. *P < 0.05; **P < 0.001

Twenty-two parturients in the esketamine group, while 37 in the control group had occurred PPD, with EPDS score > 9 (13.8% vs 23.1%, *P* = 0.0430) at 4 d after CS, however no significant difference was found at 42 d (17% vs 25%, *P* = 0.0987) (Fig. 2). PPD score at 4 d and 42 d were positive correlated (spearman *r* = 0.6025, *P* < 0.0001) (Fig. 3). Esketamine 0.2 mg/kg could reduce the NRS score at 6 h,12 h and 24 h after surgery (*P* < 0.05), while had no effect on the NRS score 48 h after surgery (*P* = 0.1730) (Fig. 4). Esketamine could reduce the use of vasoactive drugs during the operation (*P* = 0.0343) (Fig. 5). The incidences of maternal dizziness (17.0%, *P* < 0.0001), blurred vision (5%, *P* = 0.0035), illusion (3.8%, *P* = 0.0146) and drowsiness (3.8%, *P* = 0.0131) in the esketamine group were obviously higher than those in the control group (Fig. 6).

**Fig. 7:**
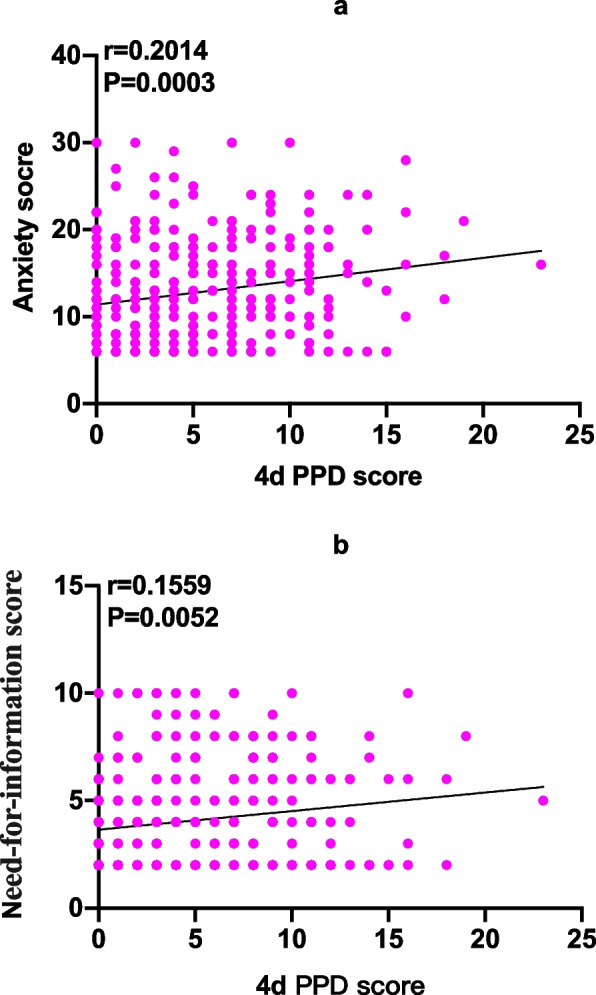
Correlations between anxiety score (**a**) and need for information score (**b**) with 4 d postpartum depression (PPD) score

Due to the skewed distribution of APAIS score and 4 d EPDS score, Spearm was used for correlation analysis, and it was found that postpartum 4 d EPDS score was positively correlated with the total APAIS score (*r* = 0.2014, *P* = 0.0003) and information need score (*r* = 0.1559, *P* = 0.0052), however the correlation coefficient was relatively low (Fig. 7).

### Further study outcome

In order to analyze the possible related factors of depression, we analyzed the following factors. The parturients with EPDS score > 9 points were included in the depressed group (*n* = 59), and the others were in the non-depressed group (*n* = 260). A secondary analysis of the data showed that the total APAIS score in the depressed group was higher than that in the non-depressed group (*P* = 0.0069), the NRS 24 h after surgery was significantly higher than that in the non-depressed group (*P* = 0.0136), and the numbers of analgesic pump compressions were also higher than that in the non-depressed group (*P* = 0.0081). There was no significant difference between the two groups in educational levels (*P* = 0.9886) and whether the fetus was delivered as expected (*P* = 0.8508) (Table 2).

**Table 2:** Basic data and intraoperative conditions of parturients in the depressed group and the non-depressed group

Variables	Depression (*n* = 59)	Non-depression (*n* = 260)	*P* value
Age (year)	30.5 ± 3.4	30.6 ± 3.9	0.8691
Height (cm)	161.4 ± 5.5	161.7 ± 4.6	0.7053
Weight (kg)	73.6 ± 10.5	74.2 ± 11.4	0.6972
BMI (kg/m2)	28.3 ± 3.9	28.4 ± 4.1	0.8500
APAIS Score			
Total score	14.6 ± 6.3	12.4 ± 5.6	0.0069
Information requirement score	4.4 ± 2.4	4.0 ± 2.4	0.2565
Education level			0.9886
Primary school	0(0)	1(0.4%)	
Secondary school	3(5.1%)	16(6.1%)	
High school	7(11.8%)	27(10.4%)	
Bachelor	43(72.9%)	185(71.2%)	
Master	5(8.5%)	25(9.6%)	
Doctor	1(1.7%)	6(2.3%)	
Employment	46(78.0%)	219(84.2%)	0.2519
Prior history of CS	17(28.8%)	77(29.6%)	> 0.9999
Type of operation			0.5565
Emergency	38(64.4%)	155(59.6%)	
Selective	21(35.6%)	105(40.4%)	
Mode of pregnancy			0.1422
Natural	58(98.3%)	240(92.3%)	
Unnatural	1(1.7%)	20(7.7%)	
Anticipate			0.8508
Expected	14(23.7%)	59(22.7%)	
Unexpected	7(11.9%)	42((16.1%)	
Indifferent	25(42.4%)	100(38.5%)	
Known	13(22.0%)	59(22.7%)	
Surgery time (minute)	57.7 ± 12.2	61.2 ± 12.8	0.0534
Anesthesia time (minute)	75.6 ± 13.0	79.1 ± 14.6	0.0825
Uncomfortable	10(17.0%)	55(21.2%)	0.5916
NRS score			
6 h	2.8 ± 0.5	2.7 ± 0.5	0.5031
12 h	2.5 ± 0.5	2.5 ± 0.6	0.6973
24 h	2.2 ± 0.6	2.0 ± 0.6	0.0136
48 h	1.5 ± 0.6	1.4 ± 0.5	0.3108
Number of pressers of PCIA	25.4 ± 16.3	18.6 ± 15.9	0.0081

Data are presented as mean ± SD, median (IQR), or number (percentage)

*BMI* body mass index, *APAIS* Amsterdam Anxiety and Information Scale, *NRS* Numeric Rating Scale, *PCIA* patient controlled intravenous analgesia, *CS* cesarean section

## Discussion

This study found that injecting 0.2 mg/kg esketamine for 40 min during cesarean section could prevent the incidence of PPD at 4 day but not 42 day. Esketamine could reduce the NRS score at 6 h, 12 h and 24 h after surgery (*P* < 0.05).

PPD is a relatively common postpartum mental disorder, with an incidence of 10%-20% in Western countries [1, 6] and a higher incidence in developing countries, about 22%-28% [3, 4]. This study found that the PPD (EPDS score > 9) incidence at 4 day was 23.1%, 25% at 42 d, which was basically consistent with previous studies.

Spinal anesthesia (SA) has become the most common anesthetic approach for cesarean section, which can reduce maternal mortality [27]. Simple technique, fast and assurance in its effect, and the influence on the fetus is small are the advantages of SA. However, post-spinal hypotension, relatively short duration,not sufficient postoperative analgesia are all the drawbacks of SA. A series of measures can compensate for the shortcomings of SA, such as neuraxial administration of opioids or a2 adrenergic receptor agonists, reducing the dosage of bupivacaine, can prolong the time for the first rescue analgesia, and reduce the occurrence of intraoperative hypotension [28, 29]. Unfortunately, these measures are not routinely performed in our hospital, so no drugs other than local anesthetics were added into the subarachnoid space in this study. In view of the same anesthesia procedure is carried out in all the parturients, it shouldn't affect the final results, although the PPD incidence would be different in other strategies.

A number of clinical studies have confirmed that subanesthetic dose of ketamine (0.5 mg/kg) has a rapid antidepressant effect on refractory depression, which can reduce the suicidal intention of patients [13, 14], and ketamine also has a preventive effect on PPD [30]. Esketamine is an enantiomide of ketamine, and its anesthetic effect is twice that of ketamine [15, 16], so we caculated the study dose ot esketamine was 0.25 mg/kg, in order to convenient for calculation and administration, we adopted 0.2 mg/kg at last. Some studies have found that administration of esketamine does not prevent the occurrence of PPD [31, 32]. However, more studies have found that either single intravenous administration of esketamine [33] or using in PCIA can reduce the occurrence of PPD [4, 34, 35]. A recent study using traditional logistic regression and machine learning models also recommended esketamine for the treatment of PPD [36]. In previous studies, esketamine was given through intravenous injection [31–33], while our study used intravenous pumping continued for 40 min, which was closer to the administration method of ketamine in antidepressant studies. Consistent with most previous studies, we found 0.2 mg/kg esketamine during cesarean section could reduce the occurrence of PPD 4 d postpartum and there was positive correlation between PPD scores at 4 d and 42 d, which suggested that the parturient with an early PPD was more likely to turn into a long-term PPD [4, 7].

There are multiple factors that contribute to the occurrence and development of PPD: a history of mental illness, depression and anxiety during pregnancy, life stress, inadequate social support, poor marital relationships, personal susceptibility and low socioeconomic status [37, 38]. A prospective study of 497 women showed that women who suffered from anxiety during pregnancy had a three-fold increased risk of intense depressive symptoms postpartum [39]. Our study also found that 4 days postpartum EPDS score was positively correlated with the total APAIS scores and information demand scores, although the correlation coefficients were low, a further analysis of the data also found that the total APAIS scores of the depressed group were higher than that of the non-depressed group, which demonstrated the correlation between maternal anxiety and PPD from another hand. In addition, literatures have shown that acute and chronic pain in the perinatal period is also an important risk factor for PPD [40–42]. This study found that the NRS score of women in the depressed group on the first day after delivery was significantly higher than that in the non-depressed group, and the numbers of analgesic pump compression were also higher than those in the non-depressed group. Meanwhile, this study found that 0.2 mg/kg esketamine could reduce acute pain at 6 h, 12 h and 24 h after surgery. According of this, we speculated that the preventive effect of esketamine on PPD might be related with or partly related with its ability to reduce postoperative pain.

It is well known that esketamine can increase blood pressure [43–45]. Due to vasodilation after intraspinal anesthesia and supine hypotension in women, vasoactive drugs were used to maintain stable maternal blood pressure. Therefore, this study did not make statistical comparisons on maternal blood pressure, but it was found that the use of positive vasoactive drugs in women in the esketamine group was indeed lower than that in the control group.

Common side effects of esketamine include dissociation symptoms, dizziness, drowsiness, nausea and vomiting, elevated blood pressure, and increased heart rate, etc., but they are usually transient, mild, and self-limiting [43–45]. In our study, esketamine 0.2 mg/kg could increase the maternal discomfort during the operation, including dizziness, blurred vision, hallucination and drowsiness. There was no statistical difference in the other side reactions, and all of which were mild and occured during administration, and usually return to normal before leaving the operation room. However, other studies suggested that esketamine did not increase maternal side effects [29, 30], this discrepancy might due to the inconsistency with the observation time and administration mode. Therefore, the effect of a smaller dose of esketamine on postpartum depression should be studied in the future to reduce maternal discomfort.

There were still several shortcomings in this study. First, the study was a single-center study, a muti-center study in the future is needed. Second, due to the long period of data collection, there might be differences in surgical and anesthesia techniques and concepts, as well as differences in the use of consumables and drug batches, but this study was a randomized controlled study, which could mitigate this confounding factor. Third, this study only studied a single dose of esketamine, and did not study other smaller doses, which might be fewer side-effects. Fourth, no other risk factors of PPD were collected, including stressful events in life, marital relationships, and economic situations, etc. Fifth, only EPDS scoring scale was used to score and diagnose PPD, and no other scoring scale was used. Although EPDS scale has been widely used, it is a relatively reliable scale for diagnosing PPD [46]. Sixth, Only PPD at 4 d and 42d were studied, while long-term PPD was not assessed.

## Conclusions

Intraoperative injection of esketamine (0.2 mg/kg/40 min) prevented PPD (EPDS score > 9) at 4 days after CS, which also reduced the NRS scores at 6 h, 12 h and 24 h after surgery, as well as the use of vasoactive drugs during surgery, but the occurrence of maternal side effects such as dizziness, blurred vision, drowsiness and hallucination were increased. Due to the side effects of esketamine, although it can reduce the occurrence of PPD, its use in the prevention of PPD needs to be cautious, and large multi-center studies are needed to investigate the effect of low doses of esketamine on PPD.

## Data Availability

The data supporting the fndings of this study are not publicly available because of institutional policy but are available from the corresponding author on reasonable request.

## Abbreviations

ASA: American Society of Anesthesiology
APAIS: Amsterdam anxiety and Information scale
BMI: Body mass index
CS: Cesarean section
EPDS: Edinburgh postnatal depression scale
NMDA: N-methyl-D-aspartate
NRS: Numeric Rating Scales
PCIA: Patient-controlled intravenous analgesia
PPD: Postpartum depression
RSS: Ramsay sedation scale
SA: Spinal anesthesia
SDS: Self-rating depression scale
